# Protein expression of c-erbB-2 and p53 in normal ducts, ductal carcinoma *in situ* and invasive carcinoma of the same breast

**DOI:** 10.1590/S1516-31802006000300002

**Published:** 2006-05-04

**Authors:** Marcus Vinicius Martins de Menezes, Anna Letícia Oliveira Cestari, Orlando Almeida, Marcelo Alvarenga, Glauce Aparecida Pinto, Maria Salete Costa Gurgel, Gustavo Antônio de Souza, Luiz Carlos Zeferino

**Keywords:** Breast neoplasms, Ductal carcinoma *in situ*, Carcinoma, Receptor erbB-2, Protein p53, Neoplasias mamárias, Carcinoma ductal *in situ*, Carcinoma de ductos infiltrante, Proteína c-erbB-2, Proteína p53

## Abstract

**CONTEXT AND OBJECTIVE:**

Breast cancer is thought to derive from progressively aberrant, non-invasive breast lesions, but it is not known exactly how invasive breast cancer develops from these lesions. The aim of this study was to verify the changes in c-erbB-2 and p53 protein expression between non-neoplastic ducts, ductal carcinoma in situ and invasive ductal carcinoma found in the same breast.

**DESIGN AND SETTING:**

This was a cross-sectional study at Centro de Atenção Integral à Saúde da Mulher, Campinas, Brazil.

**METHODS:**

Fifty-six women with invasive ductal carcinoma and ductal carcinoma *in situ* in the same breast were included. The expression of c-erbB-2 and p53 proteins was assessed in non-neoplastic and neoplastic cells using immunohistochemical techniques.

**RESULTS:**

The c-erbB-2 protein was absent in non-neoplastic ducts but was present in 46% and 36% of *in situ* and invasive carcinoma components, respectively. Only 2% of non-neoplastic ducts, and 18% and 16% of ductal carcinoma *in situ* and invasive carcinoma components, respectively, were positive for p53 protein. No significant difference in c-erbB-2 and p53 protein expression was observed between *in situ* and invasive components. The nuclear grade agreement between *in situ* and invasive carcinoma was very good.

**CONCLUSIONS:**

The invasiveness of ductal carcinoma *in situ* seems to be independent of the Her-2/neu and TP53 genes. The general features of an occurrence of breast carcinoma are formulated at the outset of carcinogenesis, and the Her-2/neu and TP53 genes are involved.

## RESUMO

**CONTEXTO E OBJETIVO:**

O câncer de mama se origina de lesões não-invasivas, tais como as hiperplasias atípicas e o carcinoma ductal *in situ*, porém não se sabe exatamente como o câncer se torna invasivo. O objetivo foi verificar alterações na expressão das proteínas c-erbB-2 e p53 entre ductos não-neoplásicos, carcinoma ductal *in situ* e carcinoma ductal invasivo presentes na mesma mama.

**TIPO DE ESTUDO E LOCAL:**

Estudo transversal, realizado no Centro de Atenção Integral à Saúde da Mulher, Campinas, São Paulo, Brasil.

**MÉTODOS:**

Cinqüenta e seis mulheres com o diagnóstico de carcinoma ductal invasivo e carcinoma ductal *in situ* na mesma mama foram selecionadas e incluídas neste estudo. A expressão das proteínas c-erbB-2 e p53 foi avaliada usando imunoistoquímica.

**RESULTADOS:**

A proteína c-erbB-2 estava ausente nos ductos não-neoplásicos, mas estava presente em 46% e 36%, respectivamente, dos componentes de carcinomas *in situ* e invasivos. Apenas 2% dos ductos nãoneoplásicos, 18% e 16% dos carcinomas *in situ* e carcinomas invasivos, respectivamente, foram positivos para a proteína p53. Não houve diferença significativa na expressão das proteínas c-erbB-2 e p53 entre os carcinomas ductal *in situ* e invasivo. A concordância do grau nuclear entre os carcinomas ductal *in situ* e invasivo foi muito boa.

**CONCLUSÕES:**

A capacidade de invadir do carcinoma *in situ* parece independente dos genes HER-2/neu e TP53. A aparência geral do carcinoma de mama é formulada na iniciação da carcinogênese e os genes Her-2/neu e TP53 estão envolvidos.

## INTRODUCTION

Breast cancer is thought to derive from progressively aberrant, non-invasive breast lesions such as atypical hyperplasia and ductal carcinoma *in situ*, but it is not known exactly how invasive breast cancer develops from these lesions. Chromosomal imbalances occur, with gain or loss at multiple *loci*, as hyperplastic lesions progress from ductal carcinoma *in situ* to invasive breast cancer.^1,2^ Nevertheless, the presence of shared chromosomal changes in both ductal carcinoma *in situ* and synchronous, adjacent invasive cancers demonstrates their clonal, evolutionary relationship.^3^

HER-2/neu and TP53 gene expression in normal breast tissue is different from what is found in invasive breast carcinoma, and this variation can be assessed by immunohistochemistry.^4,5^ Many studies have already analyzed c-erbB-2 and p53 protein expression in ductal carcinoma *in situ* and invasive ductal carcinoma, but most of these studies were carried out in tissue from different women, thereby restricting the usefulness of these data for studying tumor progression.^4,5^

Progression from ductal carcinoma *in situ* to invasive carcinoma occurs at specific points within the preexisting *in situ* lesion, and therefore ductal carcinoma *in situ* and invasive ductal carcinoma are frequently found in the same breast.^6^ We made the assumption that such cases would be a good model in which to study the relationship between non-neoplastic ducts, ductal carcinoma *in situ* and invasive ductal carcinoma.

## OBJECTIVE

The aim of this study was to verify the changes in protein expression between nonneoplastic ducts, ductal carcinoma *in situ* and invasive ductal carcinoma found in the same breast.

## MATERIALS AND METHODS

Ninety-eight women diagnosed with invasive ductal carcinoma and ductal carcinoma *in situ* in the same breast were selected consecutively for this study. The patients were seen in our service (Centro de Atenção Integral à Saúde da Mulher, CAISM) or in a private medical service in Campinas, São Paulo, Brazil, between 1994 and 1999. The paraffin-embedded blocks were sectioned and the slides were stained with hematoxylin-eosin to identify the tissue areas in which non-neoplastic ducts, ductal carcinoma *in situ* and invasive ductal carcinoma were found. Forty-two of these women were excluded from the study because the remaining breast tissue in the paraffin blocks was insufficient for immunohistochemical analysis. The remaining fifty-six women were enrolled in the study: thirty-eight of them were diagnosed as having invasive ductal carcinoma in clinical stage I and eighteen in stage II. Less prevalent histological types were not included, in order to obtain a homogeneous sample.

The same pathologist performed the patho-anatomical analyses and established the final histological diagnosis. Ducts were defined as non-neoplastic when they were normal ducts (one layer of cells) or presented typical ductal hyperplasia (up to four layers of cells without atypia). The nuclear grade of tissue components was also evaluated and classified according to the 1997 Consensus Conference on the Classification of Ductal Carcinoma *In Situ*.^7^

The expression of c-erbB-2 and p53 proteins was assessed using immunohistochemistry. Specific monoclonal primary antibodies for c-erbB-2 (RxH, Dako, code A0485-1, at 1:300 dilution) and for p53 (MxH, clone D07, Dako, code M7001-1, at 1:100 dilution) were used, and steam heating was used to unmask the antigens. The slides were incubated using Envision labeled polymer components (Dako, code K1491). Development was carried out using DAB (3-3’-diaminobenzidine, Sigma, code D5637). All immunohistochemical assays were performed using external positive controls: invasive ductal carcinoma for c-erbB-2 and borderline ovarian tumor for p53.

Immunohistochemical analysis was carried out using 40x magnification, by a single pathologist who was blinded to the results from the pathoanatomical analyses. The c-erbB-2 protein expression was considered positive when more than 10% of the cells were stained, and p53 protein expression was considered positive when more than 1% of the nuclei were stained, regardless of the intensity of the staining.

Odds ratios were used to evaluate the strength of the association between pairs of variables, and the 95% confidence intervals (CI) were calculated. The kappa coefficient was used to verify the agreement between the nuclear grade of ductal carcinoma *in situ* and the nuclear grade of invasive ductal carcinoma in the same breast, and was classified as: poor (< 0.20); fair (0.21-0.40); moderate (0.41-0.60); good (0.61-0.80) or very good (0.81-1.00).^8^ For statistical purposes, nuclear grades 1 and 2 were analyzed together.

## RESULTS

The c-erbB-2 protein was absent from nonneoplastic ducts but was present in 46% of the cases of ductal carcinoma *in situ*, resulting in an odds ratio of 98.18 (95% CI: 5.78-1667.60). There was no statistical difference in c-erbB-2 expression between *in situ* and invasive components. Only 2% of the non-neoplastic ducts and 18% of the ductal carcinoma *in situ* components showed p53 protein expression, resulting in an odds ratio of 11.96 (95% CI: 1.47-96.92), while the difference in p53 positivity between ductal carcinoma *in situ* and invasive ductal carcinoma gave a nonsignificant result (Table 1). Every case with positive c-erbB-2 expression in the invasive carcinoma component showed also positive expression in the *in situ* component. Eight out of the nine cases with positive p53 expression in the invasive component also showed positive expression in the *in situ* component (data not shown).

**Table 1.** Association of protein expression of c-erbB-2 and p53 in non-neoplastic ducts, ductal carcinoma *in situ* and invasive ductal carcinoma of the same breast

Protein expression	Non-neoplastic ducts		Ductal carcinoma *in situ*		Invasive ductal carcinoma
	n (%)		n (%)		n (%)
c-erbB-2 positive	0 (0)		26 (46)		20 (36)
c-erbB-2 negative	56 (100)		30 (54)		36 (64)
OR (95% CI)*		98.2 (5.8-1667.6)		0.6 (0.3-1.4)	
p53 positive	1 (2)		10 (18)		9 (16)
p53 negative	55 (98)		46 (82)		47 (84)
OR (95% CI)*		12.0 (1.5-96.9)		0.9 (0.3-2.4)	
**Total cases**	**56 (100)**		**56 (100)**		**56 (100)**

*OR = odds ratio; 95% CI = 95% confidence interval.*

* *= Both OR included data referring to ductal carcinoma in situ.*

Positive c-erbB-2 protein expression was associated with nuclear grade 3 in both the *in situ* and the invasive components of ductal carcinoma. The p53 protein expression showed a similar association (Table 2). There were no cases of invasive ductal carcinoma with a higher nuclear grade than what was found in the ductal carcinoma *in situ* component, and in 52/56 cases both components were found to have the same nuclear grade. The kappa coefficient was 0.88 (0.77-0.99), thus indicating very good agreement (Table 3).

**Table 2.** Association of c-erbB-2 and p53 protein expression according to nuclear grade of ductal carcinoma *in situ* and invasive ductal carcinoma of the same breast

Protein expression	Ductal carcinoma *in situ*			Invasive ductal carcinoma		
	NG 1 or 2 n(%)	NG 3 n(%)	OR (95% CI)	NG 1 or 2 n(%)	NG 3 n(%)	OR (95% CI)
c-erbB-2 positive	8 (27)	18 (69)	6.2 (2.0-19.8)	6 (18)	14 (61)	7.0 (2.1-23.7)
p53 positive	2 (7)	8 (31)	6.2 (1.2-32.7)	1 (3)	8 (35)	17.1 (2.0-149.1)
**Total cases** *	**30 (100)**	**26 (100)**		**33 (100)**	**23 (100)**	

*OR = odds ratio; 95% CI = 95% confidence interval; NG = nuclear grade.*

* *Positive plus negative protein expression cases.*

**Table 3.** Distribution of cases according to the nuclear grade of ductal carcinoma *in situ* and invasive ductal carcinoma of the same breast: analysis of nuclear grade agreement

Invasive ductal carcinoma	Ductal carcinoma in situ			Total
	NG 1	NG 2	NG 3	n (%)
NG 1	6	1	0	7 (12.5)
NG 2	0	23	3	26 (46.4)
NG 3	0	0	23	23 (41.1)
**Total**	**6 (10.7)**	**24 (42.9)**	**26 (46.4)**	**56 (100)**
**Kappa coefficient (95% CI): 0.88 (0.77-0.99)**				

*NG = nuclear grade; 95% CI = 95% confidence interval.*

## DISCUSSION

The expression of c-erbB-2 and p53 proteins showed a large variation between the non-neoplastic ducts and ductal carcinoma *in situ* components, but most of the cases showed very similar protein expression and good nuclear grade agreement between ductal carcinoma *in situ* and invasive ductal carcinoma components.

C-erbB-2 protein expression has not been found in either normal breast tissue or ductal hyperplasia, and there is a broad consensus regarding these results.^9–13^ A few studies have reported expression of this protein in rare cases of atypical ductal hyperplasia,^14-16^ and there could be two possible explanations for these findings. First, these rare cases could be genetically different and, second, the interobserver reproducibility of borderline lesions with these diagnostic criteria is poor. Atypical ductal hyperplasia with c-erbB-2 positive expression may have been classified as well-differentiated ductal carcinoma *in situ* by other observers.^17^ Few cases of well-differentiated ductal carcinoma *in situ* show c-erbB-2 protein expression.^3,5^

A review^18^ has shown that overall c-erbB-2 positivity for all types of ductal carcinoma *in situ* ranges between 21% and 64%, and for comedo ductal carcinoma *in situ* between 62% and 81%. The positivity is lower in cases of invasive ductal carcinoma, ranging between 16% and 40%,^18^ and these data are in agreement with the results from the present study. There is also a clear association between c-erbB-2 positivity and worse nuclear and histological grades, tumor aneuploidy and high rate of proliferation, and this is more frequent in ductal carcinoma *in situ* than in invasive ductal carcinoma.^5,18,19^ This profile for c-erbB-2 protein expression is quite similar to the p53 protein expression profile.^20,21^

The morphological features and immunohistochemical profile of the ductal carcinoma *in situ* and invasive ductal carcinoma components of the same specimens have been found to be very similar.^5,22^ Cases of ductal carcinoma *in situ* have shown a more “malignant picture” than cases of invasive cancer.^5,19^ In the present study, fifty-two out of fifty-six cases showed ductal carcinoma *in situ* and invasive ductal carcinoma components with the same nuclear grade, while in the other four cases, more malignant features were found in the ductal carcinoma *in situ* component. This may suggest that undifferentiation and invasiveness of the ductal carcinoma *in situ* are not necessarily associated. Similar results were obtained by Warnberg et al.^5^ when comparing the nuclear grades of ductal carcinoma *in situ* and invasive ductal carcinoma in the same breast. There was concordance between the nuclear grades of ductal carcinoma *in situ* and invasive ductal carcinoma in 102 cases out of 259. In 151 cases, the nuclear grade was higher in ductal carcinoma *in situ* than in invasive ductal carcinoma, and in only six cases was the nuclear grade of invasive ductal carcinoma higher than that of ductal carcinoma *in situ*. These data suggest that the degree of aggressiveness of the tumor, i.e. its prognosis, is genetically “formulated” at the beginning of carcinogenesis and is maintained throughout the evolution of the disease.

A study of gene expression patterns in ductal carcinoma *in situ* and invasive ductal carcinoma, carried out using serial analysis of gene expression (SAGE), found that the most dramatic transcriptome change occurs at the time of transition from normal to ductal carcinoma *in situ*, when there is no clear universal “*in situ*” or “invasive” molecular signature of the tumor. That study suggested that some genes may be able to define biologically and clinically meaningful subgroups of ductal carcinoma *in situ* with a high risk of progression to invasive disease.^23^

Another study using microdissection and DNA microarrays revealed extensive similarities at the transcriptome level among the distinct stages of progression and suggested that gene expression alterations that conferred the potential for invasive growth are already present in the preinvasive stages. In contrast, different tumor grades are associated with distinct gene expression signatures. Furthermore, a subset of genes associated with high tumor grade is quantitatively correlated with the transition from preinvasive to invasive growth.^24^

Atypical ductal hyperplasia is an early aberrant breast lesion that may progress to low nuclear grade ductal carcinoma *in situ*, which would continue accumulating genetic alterations^13^ and could lead progressively to intermediate and high nuclear grade ductal carcinoma *in situ*.

Considering the findings of these studies, the ductal carcinoma *in situ* cells could acquire the ability to cross the basal membrane and trigger an invasive ductal carcinoma with very similar morphological and immunohisto-chemical features. Therefore, a low-grade ductal carcinoma *in situ* could trigger a low-grade invasive ductal carcinoma and the same could occur in cases of intermediate and high-grade tumors. The *in situ* ductal carcinomas that do not become invasive at an early stage would reach high nuclear grade and would proliferate along the mammary duct, thus accumulating areas of necrosis and calcification.

There are two possible explanations for the few cases in our study in which the ductal carcinoma *in situ* component showed a higher nuclear grade than did the invasive ductal carcinoma component. First, the cellular clone that becomes invasive may be more differentiated than the other clones present in the ductal carcinoma *in situ* component. Second, after invasion has occurred, the remaining ductal carcinoma *in situ* component would continue accumulating genetic alterations, thereby reaching higher nuclear grades than the cellular clone from which the invasive ductal carcinoma originated.

In short, the results from our study suggest that the Her-2/neu and TP53 genes are likely to be involved in the beginning of breast carcinogenesis (induction) and undifferentiation of ductal carcinoma *in situ*, but not in the progression from ductal carcinoma *in situ* to invasive carcinoma. We must emphasize that the variation in c-erbB-2 protein expression in the three components of the same specimens is due exclusively to local modifications of ductal cells, since there are no genetic differences other than those acquired over the course of the evolution of carcinogenesis. Studies carried out on ductal carcinoma *in situ* and invasive ductal carcinoma in different women are biased by individual genetic differences.

## CONCLUSIONS

The invasiveness of ductal carcinoma *in situ* seems to be independent of the Her-2/neu and TP53 genes. Our results suggest that the general features of breast carcinoma occur-rences are formulated at the outset of carcinogenesis, and that the Her-2/neu and TP53 genes are involved in this. The morphological features and immunohistochemical profile of the ductal carcinoma *in situ* and invasive ductal carcinoma components of the same specimens are very similar.
